# Synthesis of New Liquid Crystalline Diglycidyl Ethers

**DOI:** 10.3390/molecules17010645

**Published:** 2012-01-10

**Authors:** Issam Ahmed Mohammed, Rashidah Mohamed Hamidi

**Affiliations:** School of Industrial Technology, University Sains Malaysia, Penang 11800, Malaysia

**Keywords:** synthesis, diglycidyl ether, schiff base, liquid crystal

## Abstract

The phenolic Schiff bases **I**–**VI** were synthesized by condensation reactions between various diamines, namely *o*-dianisidine, *o*-tolidine and ethylenediamine with vanillin or *p*-hydroxybenzaldehyde and subsequent reactions between these phenolic Schiff bases and epichlorohydrin to produce new diglycidyl ethers **Ia**–**VIa**. The structures of these compounds were confirmed by CHN, FT-IR, ^1^H-NMR, and ^13^C-NMR spectroscopy. Their thermotropic liquid crystalline behavior was studied using differential scanning calorimetry (DSC) and polarizing optical microscopy (POM). All the diglycidyl ethers prepared exhibit nematic mesophases, except for **Va** and **VIa**, which did not show any transition mesophases, but simply flow to liquids.

## 1. Introduction

Diglycidyl ether can be differentiated from other organic compounds by the presence of two terminal oxirane groups, which are able to react with compounds possessing active hydrogen atoms, including amines [1], amides or mercaptans [2] and alcohols [3,4]. Glycidyl ethers have been widely used since the late 1940s as basic components of epoxy resins [5]. Many research studies involving this particular material (glycidyl ether) and leading to the formation of diol glycidyl ether bridged-cyclens [6], epoxy resins based on recycled poly(ethylene terephtalate) (PET) [7], and oxalolidinones from epoxide compounds [8,9] have been reported, among others. Each of the aforementioned compounds was made for various applications such as gene delivery, organic coatings, and drug development, respectively. However, there are few articles that describe organic materials, e.g., liquid crystals, based on glycidyl ether-containing Schiff base groups. Liquid crystals are organic materials possessing unique direction-dependent properties due to the fact that the molecules are anisotropically (directionally) oriented. Such materials have potential applications as polarizers, liquid crystal displays and optoelectronic devices [10]. Rapid developments in liquid crystal research has resulted in the emergence of many other applications, for instance organic light emitting diodes, photovoltaic devices, organic field effect transistors, gas sensors, *etc.* [11]. Schiff base groups are typically formed by the condensation of a primary amine and an aldehyde [12], and these species are also known to be important intermediates for the synthesis of various bioactive compounds [13]. In this study, we synthesized new liquid crystalline diglycidyl ethers with Schiff base groups where we predicted that the compounds produced might exhibit good thermal stability and many desirable properties due to the resonance stabilization of the poly-Schiff’s base unit [14].

## 2. Results and Discussion

### 2.1. Characterization of the Phenolic Schiff Bases **I–VI**

The preparation of the phenol Schiff base compounds is described in the Experimental section. Elucidation of the chemical structure of each sample prepared was carried out by means of elemental analysis (CHN), FT-IR, ^1^H-NMR and ^13^C-NMR. Structures of resulting compounds were first confirmed by FT-IR spectroscopy which plays a vital role in verifying the existence of the typical key functional groups. In this case the infrared spectra displayed characteristic bands in the 1,620–1,634 cm^−1^ region attributed to the presence of the Schiff base group (-CH=N), hence also proving the completion of the reaction between the NH_2_ and C=O groups. The band near 3,350–3,414 cm^−1^ was assigned to the OH group, while the ones at 1,502–1,516 and 1,576–1,592 correspond to the aromatic -C=C-. Data on elemental analysis, ^1^H-NMR and ^13^C-NMR which support the formation of the expected compounds are included in the Experimental section.

### 2.2. Characterization of the Diglycidyl Ethers **Ia–VIa**

**Figure 1:**
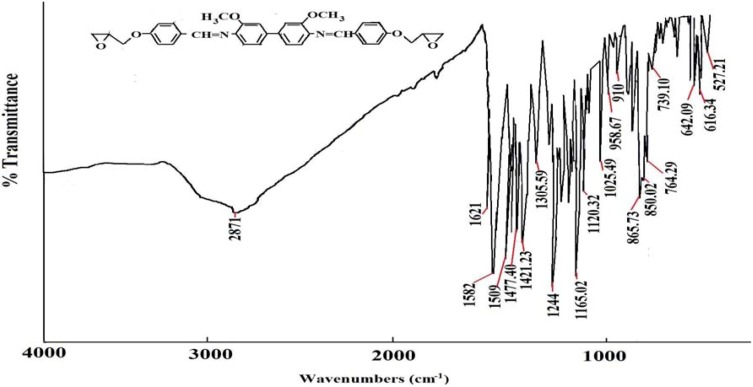
FT-IR spectrum of compound **IIa**.

**Figure 2:**
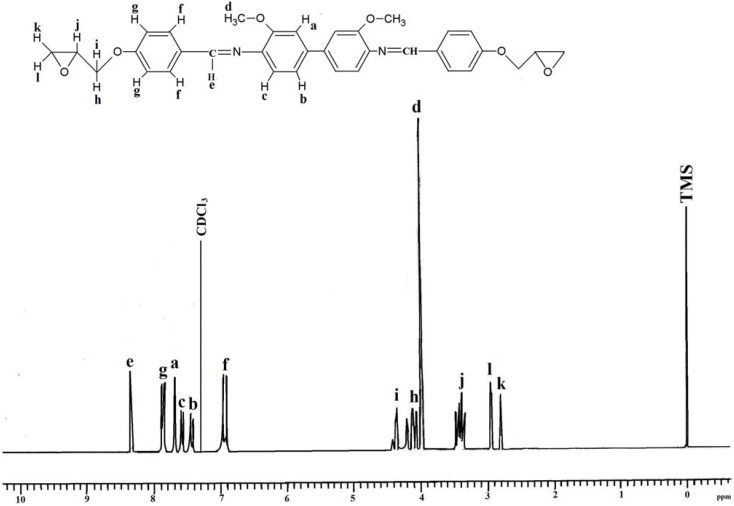
^1^H-NMR spectrum of **IIa** in CDCl_3_.

**Figure 3:**
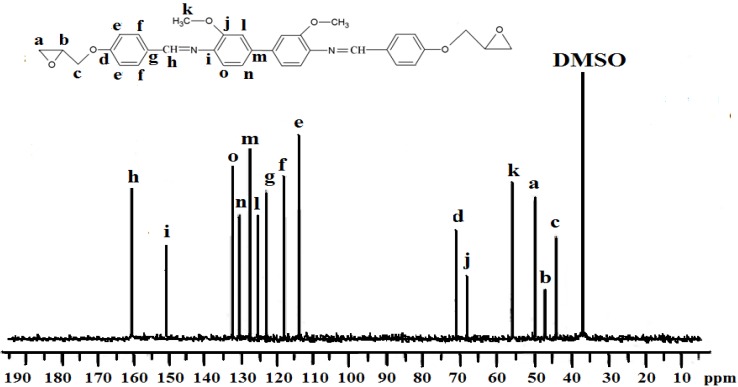
^13^C-NMR spectrum of **IIa** in DMSO-d6.

Spectroscopic measurements (FTIR, ^1^H-NMR, and ^13^C-NMR) and elemental analysis were performed to determine the identity and purity of the diglycidyl ethers **Ia**–**VIa**. An absorbance band in the 1,608 cm^−1^ to 1,624 cm^−1^ range was due to the presence of -CH=N [15], while the absorption peaks at 1,502–1,516 cm^−1^ and 1,572–1,590 cm^−1^ were due to the aromatic ring skeletal vibrations and C=C bond stretching, respectively. The whole ring stretching and the terminal oxirane rings can be observed at 910–915 cm^−1^ and 1,244–1,269 cm^−1^. Figure 1 displays the FT-IR spectrum of compound **IIa**, where in the region of 1,621 cm^−1^ (CH=N), 1,509 and 1,582 (aromatic C=C stretch) and 1,244 and 910 (oxirane ring) one may observe peaks confirming the complete formation of the respective compound. In addition, based on the ^1^H-NMR spectrum depicted in Figure 2, the presence of the oxirane ring was confirmed by the peaks appearing at 2.9–3.05 ppm as two doublets and at 3.49–3.57 ppm as multiplet. Two characteristic singlet peaks centered at 3.86 and 8.33 ppm [16] were due to the methoxy protons (-OCH_3_) and the Schiff base protons (CH=N), respectively. A multiplet peak in the 7.05–7.85 ppm range was attributed to the protons in the aromatic rings. The presence of the diglycidyl ether CH_2_ was verified from the appearance of a multiplet peak in the 4.02–4.40 range. Determination of carbons in the chemical structure was done by ^13^C-NMR and the spectrum is displayed in Figure 3. Significant peaks indicating the formation of the compound were detected at the 161.25 and 44.63–51.03 ppm regions, associated to the presence of carbons of the azomethine groups and oxirane ring, respectively. All the spectroscopic and elemental analysis results given in the Experimental section conformed the chemical structures, proving the successful formation of the target compounds.

### 2.3. Properties of the Diglycidyl Ethers **Ia–VIa**

The thermotropic liquid crystalline behavior of the diglycidyl ethers **Ia**–**VIa** was investigated by DSC and further confirmed by polarized optical microscopy. The melting (Tm) and isotropization (Ti) points were detected from the DSC thermograms for all diglycidyl ethers, except for **Va** and **VIa** which did not show any transition temperature. The DSC and POM results were both consistent and the data obtained are summarized in Table 1. Upon observation on the POM, which was equipped with a heating stage, only compounds **Ia**–**IVa** exhibited liquid crystalline behavior, whereas no trace of mesophase transition was displayed for **Va** and **VIa**, which simply flow to liquid. Figure 3 shows polarizing optical microphotographs of **Ia**–**IVa** in which all of the compounds show nematic texture. The azomethine (CH=N) linking group connecting the two core groups provides a stepped core structure, yet it maintains molecular linearity, hence providing higher stability and enabling mesophase formation [17]. According to Sun [18] compounds with higher axial ratio molecules could form a mesophase. Most thermotropic liquid crystals are calamitic molecules having a rigid core composed of two or more aromatic rings and one or more flexible terminal chains [19]. In case of **Va** and **VIa** both of them possess rigid cores and two aromatic rings in their structures, but due the insufficient rigidity, caused by the presence of a flexible chain which will interrupt the conjugation (resonance), this leads to less delocalization and hence the failure to display liquid crystal behavior. Besides determining the liquid crystalline behavior, DSC was done to analyze the thermal behavior of the compounds. It was observed that compound without -OCH_3_ substituent attached to the central aromatic ring system show higher thermal properties. This is because the presence of the methoxy group had caused thermal suppression of the molecule [20], thus lowering the melting temperature of the compounds. Moreover that substituent is capable of reducing the coplanarity of adjacent mesogenic groups and increases the diameter or decreases the axial ratio of the mesogens [21]. Different approaches were taken by other researchers, where they utilized various kinds of substituent in order to reduce the melting temperature (T_m_) as well as the isotropization temperature (T_i_).

**Table 1:** Transition point of **Ia**–**VIa**.

Diglycidyl ethers	Cr-N (°) *	N-I (°) *	∆T/°C	Transition temperatures, DSC (°)	
				Tm	Ti
**Ia**	158.0	251.0	148.0	149.8	257.0
**IIa**	160.1	275.2	115.1	157.0	285.1
**IIIa**	135.0	174.0	42.0	136.0	176.0
**IVa**	147.0	215.0	79.0	145.0	220.0
**Va**	-	-	-	138.0	-
**VIa**	-	-	-	140.0	-

Cr = crystal, N = Nematic phase, I = isotropic, * = displayed by POM.

**Figure 4:**
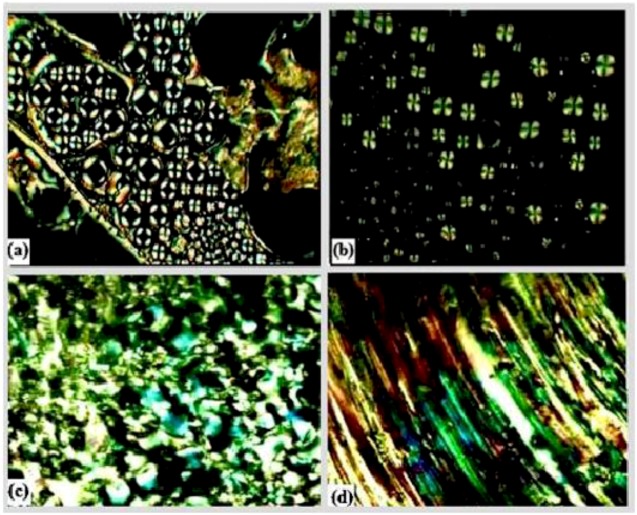
Polarized optical microscope (POM) images of (a) **Ia**, (b) **IIa**, (c) **IIIa**, and (d) **IVa**.

## 3. Experimental

### 3.1. Materials

All chemicals were commercially available and either used after standard purification or without further purification. *o*-Dianisidine, *o*-tolidine and vanillin were from Fluka (Germany), *p*-hydroxybenzaldehyde from Merck (Germany), ethylenediamine from Sigma Aldrich (Germany) epichlorohydrin from Fisher Chemicals (UK), 99.5% ethanol from Systerm® (Malaysia) and 1-butanol from R&M Chemicals (Malaysia).

### 3.2. Instruments

The FT-IR spectra were measured using a Perkin-Elmer 2000 FTIR with a potassium bromide (KBr) beam splitter. Thirty-two (32) scans were collected between 4,000 cm^−1^ and 400 cm^−1^ with a resolution of 2.0 cm^−1^. The ^1^H-NMR and ^13^C-NMR spectra were obtained using a Bruker 400 MHz spectrometer and tetramethylsilane (TMS) was used as the internal reference. CHN microanalyses were performed using a Perkin Elmer 2400 LS Series CHNS=O analyzer. Differential scanning calorimetry (DSC) studies were carried out with a Perkin-Elmer DSC7 series at a heating rate of 10 °C/min in nitrogen; melting points of LC diglycidyl ethers were obtained based on the peak of the melting temperatures from DSC analysis. Polarizing optical microscope (POM), a Carl Zeiss Axioskop equipped with 40 Linkam LTS350 hot stage, Linkam TMS94 temperature controller and Linkam LNP cooling system (pump) was used to determine the liquid crystalline mesophases.

### 3.3. General Procedure for the Preparation of the Phenolic Schiff Bases **I–VI**

**Scheme 1:**
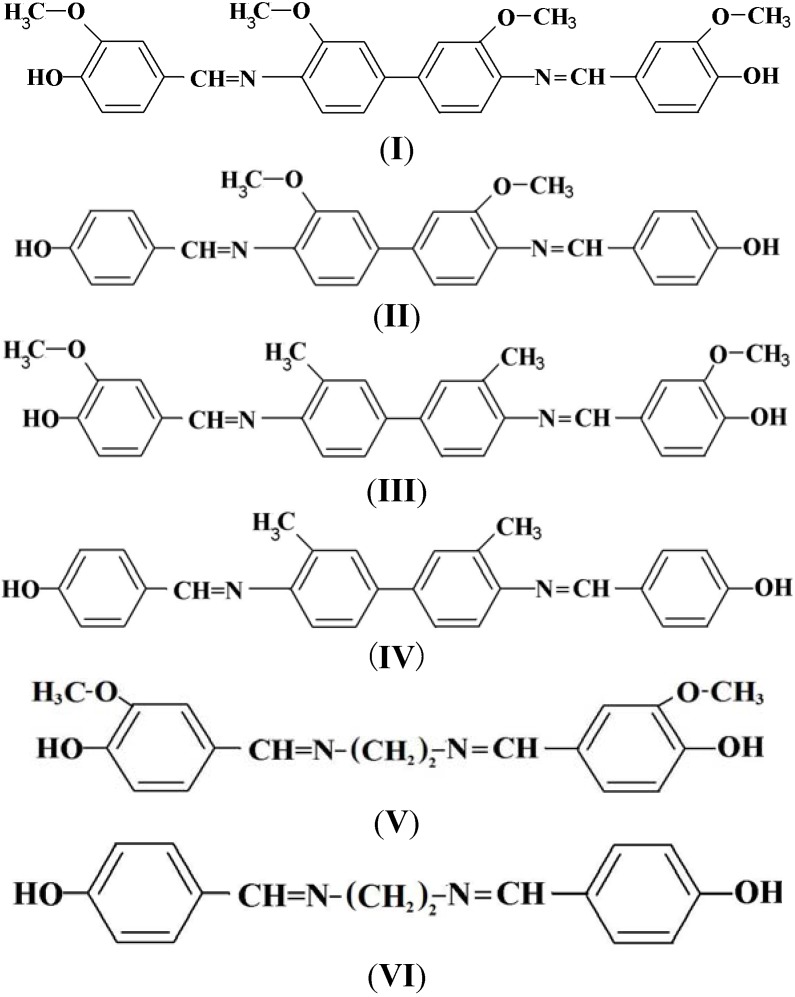
Chemical structures of the Phenolic Schiff Bases (**I**–**VI**).

A solution of aldehyde (0.02 mol) in absolute ethanol (20 mL) was added dropwise into a solution of diamine (0.01 mol) in absolute ethanol (20 mL) contained in a 500 mL reaction flask. The mixture was refluxed for 6 h with magnetic stirring. The product was then filtered, washed several times with diethyl ether and dried in a vacuum oven at 70 °C for 24 h. The chemical structures of the Phenolic Schiff Bases **I**–**VI** can be referred in Scheme 1.

*N,N’-bis(4-Hydroxy-3-methoxybenzylidene)-o-dianisidine* (**I**). Final purification was carried out by re-crystallization from acetonitrile to give a brownish powder in 70% yield. FT-IR (KBr disc): 3,369 cm^−1^ (O-H stretch), 1,622 cm^−1^ (CH=N stretch) 1,587 and 1,516 cm^−1^ (aromatic C=C stretch), 1,029 cm^−1^ (-OCH_3_ stretch). ^1^H-NMR (CDCl_3_ ppm): δ_H_9.75 (s, -OH), 8.40 (s, CH=N), 7.71, 7.62, 7.51, 7.32, 6.93 and 6.91 (s, d, d, d, s, and d aromatic protons, respectively) and 3.91 (s, -OCH_3_). ^13^C-NMR (DMSO-d_6_ ppm) δ_C_ 163.39 (CH=N), 162.71, 154.00, 153.95, 139.00, 135.29, 134.71, 133.23, 128.35, 127.77, 126.32, 117.22, 104.41 (aromatic carbons) and 54.1 (-OCH_3_). Elemental analysis: found: C, 70.45; H, 5.24; N, 5.33, C_30_H_28_N_2_O_6_. Calcd: C, 70.29; H, 5.51; N, 5.47.

*N,N’-bis(4-Hydroxybenzylidene)-o-dianisidine* (**II**). Final purification was carried out by re-crystallization from acetonitrile to give a yellowish powder in 94% yield. FT-IR (KBr disc): 3,350 cm^−1^ (O-H stretch), 1,620 cm^−1^ (CH=N stretch), 1,584 and 1,515 cm^−1^ (aromatic C=C stretch) 1,029 cm^−1^ (-OCH_3_ stretch). ^1^H-NMR (CDCl_3_ ppm): δ_H_ 10.08 (s, -OH), 8.43 (s, CH=N), 7.7, 7.54, 7.5, 7.32 and 7.07 (s, d, d, d and d aromatic protons, respectively) and 3.91 (s, -OCH_3_). ^13^C-NMR (DMSO-d_6_ ppm): δ_C_ 162.25 (CH=N), 161.10, 156.70, 141.28, 138.11, 135.69, 132.21, 128.83, 125.51, 118.00, 114.48 (aromatic carbons) and 55.4 (-OCH_3_). Elemental analysis: found: C, 73.97; H, 5.30; N, 6.44, C_28_H_24_N_2_O_4_. Calcd: C, 74.31; H, 5.35; N, 6.19.

*N,N’-bis(4-Hydroxy-3-methoxybenzylidene)-o-tolidine* (**III**). Final purification was carried out by re-crystallization from 1-butanol to give a yellow powder in about 71% yield. FT-IR (KBr disc): 3,391 cm^−1^ (O-H stretch), 1,621 cm^−1^ (CH=N stretch), 1,510 and 1,592 cm^−1^ (aromatic C=C stretch) and 1,033 cm^−1^ (-OCH_3_ stretch). ^1^H-NMR (CDCl_3_ ppm): δ_H_ 8.32 (s, CH=N), 7.99, 7.89, 7.71, 7.5, 6.88 and 6.85 (s, d, d, s and d aromatic protons respectively), 6.0 (s, -OH), 4.00 (s, -OCH_3_) and 2.45 (s, ph-CH_3_). ^13^C-NMR (DMSO-d_6_ ppm): δ_C_ 160.1 (CH=N), 157.39, 156.27, 139.82, 135.20, 131.00, 130.54, 129.45, 129.01, 128.43, 127.70, 118.22, 109.03 (aromatic carbons), 55.82 (O-CH_3_) and 18.52 (ph-CH_3_). Elemental analysis: found: C, 75.32; H, 6.07; N, 5.67, C_30_H_28_N_2_O_4_. Calcd: C, 74.97; H, 5.88; N, 5.83.

*N,N’-bis(4-Hydroxybenzylidene)-o-tolidine* (**IV**). Final purification was carried out by recrystallization from 1-butanol to give a yellow powder in 74% yield. FT-IR (KBr disc): 3,414 cm^−1^ (O-H stretch), 1,623 cm^−1^ (CH=N stretch), 1,511 and 1,576 cm^−1^ (aromatic C=C stretch). ^1^H-NMR (CDCl_3_ ppm): δ_H_ 8.56 (s, CH=N), 6.05 (s, -OH), 7.76, 7.69, 7.52, 7.3 and 6.9 (s, d, d, d and d aromatic protons, respectively) and 2.34 (s, ph-CH3). ^13^C-NMR (DMSO-d_6_ ppm): δ_C_ 159.95 (CH=N), 157.00, 141.83, 138.04, 132.87, 131.24, 129.58, 127.75, 125.97, 122.28, 116.41 (aromatic carbons) and 19.7 (ph-CH_3_). Elemental analysis: found: C, 79.49; H, 5.83; N, 6.61, C_28_H_24_N_2_O_2_. Calcd: C, 79.97; H, 5.76; N, 6.67.

*N,N’-bis(4-Hydroxy-3-methoxybenzylidene)-1,2-diaminoethane* (**V**). Final purification was carried out by recrystallization from ethanol to give a brownish powder. The yield was 72%. FT-IR (KBr disc): 3,377 cm^−1^ (O-H stretch), 1,632 cm^−1^ (CH=N stretch), 1,503 and 1,577 cm^−1^ (aromatic C=C stretch), 1,028 cm^−1^ (-OCH_3_). ^1^H-NMR (CDCl_3_ ppm): δ_H_ 9.20 (s, -OH), 8.17 (s, CH=N), 7.3, 7.12 and 6.77 (s, d and d aromatic protons, respectively), 3.77 (s, -OCH_3_) and 3.45 (s, N-CH_2_). ^13^C-NMR (DMSO-d_6_ ppm): δ_C_ 164.1 (CH=N), 56.0 (-OCH_3_), 158.04, 155.51, 130.16, 126.23, 119.31, 105.33 (aromatic carbons) and 29.27 (N-CH_2_). Elemental analysis: found: C, 65.43; H, 6.06; N, 8.44, C_18_H_20_N_2_O_4_. Calcd: C, 65.83; H, 6.14; N, 8.53.

*N,N’-bis(4-Hydroxybenzylidene)-1,2-diaminoethane* (**VI**)*.* Final purification was carried out by re-crystallization from benzene to give a brownish powder. The yield obtained was 74%. FT-IR (KBr disc): 3,363 cm^−1^ (O-H stretch), 1,634 cm^−1^ (CH=N stretch), 1,502 and 1,586 cm^−1^ (aromatic C=C stretch).^1^H-NMR (CDCl_3_ ppm): δ_H_ 9.37 (s, -OH), 8.32 (s, -CH=N), 7.25 and 6.90 (d and d, aromatic protons) and 3.78 (s, N-CH_2_).^13^C-NMR (DMSO-d_6_ ppm): δ_C_ 161.9 (CH=N), 158.55, 128.00, 124.32, 118.22, (aromatic carbons) and 26.31 (N-CH_2_). Elemental analysis: found: C, 71.39; H, 6.09; N, 10.50, C_16_H_16_N_2_O_2_. Calcd: C, 71.61; H, 6.01; N, 10.45

### 3.4. General Procedure for the Preparation of the Diglycidyl Ethers **Ia–VIa**

**Scheme 2:**
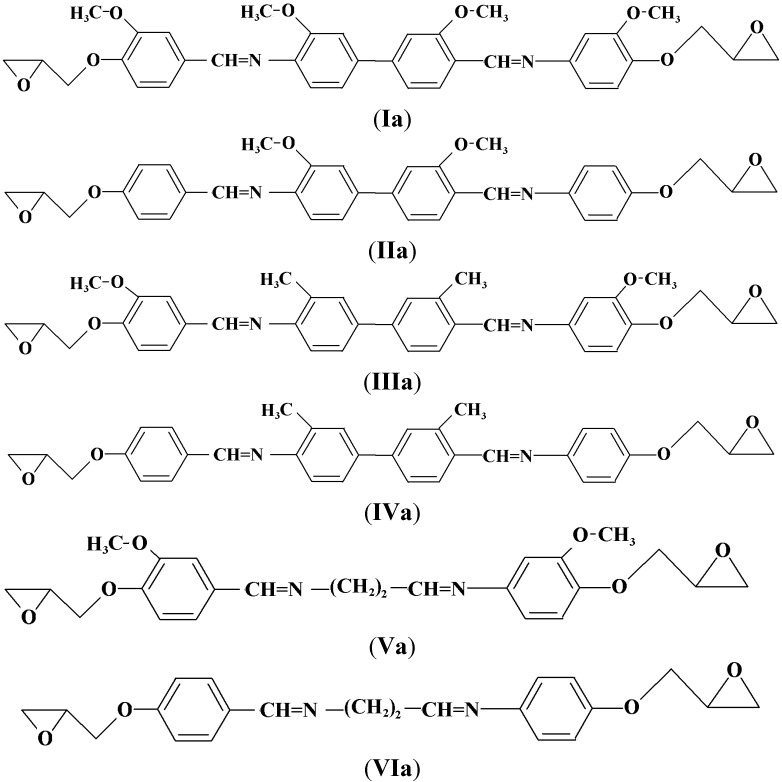
Chemical structures of the Diglycidyl Ethers (**Ia**–**VIa**).

Compounds **I**–**VI** (0.01 mol) were reacted with epichlorohydrin (0.04 mol) in a 500 mL reaction flask and the mixture was refluxed at 112 °C to promote ring closure. Then, a catalytic amount of tetrahexylammonium bromide (0.00312 mol) was added into the mixture which was heated for another 1 h. The precipitation was filtered, washed several times with diethyl ether and dried in a vacuum oven at 80 °C for 12 h. Final purification was carried out by recrystallization from toluene and the pure compounds were dried in a vacuum at 90 °C for 24 h. Scheme 2 displays the chemical structures of the Diglycidyl Ether **Ia**–**VIa**.

*3,3’-Dimethoxy-4,4’-di(2,3-epoxypropoxy-N-benzylidine)-o-dianisidine* (**Ia**). The yield of brownish powdery compound was 73%. FT-IR (KBr disc): 1,623 cm^−1^ (CH=N stretch), 1,502 and 1,588 cm^−1^ (aromatic C=C stretch), 1,247 and 915 cm^−1^ (oxirane ring). ^1^H-NMR (CDCl_3_ ppm): δ_H_ 8.22 (s, CH=N), 7.85, 7.72, 7.69, 7.34, 7.12 and 7.05 (s, d, d, d, s and d aromatic protons respectively), 4.03 (s, -OCH_3_) 2.85–2.91 and 3.39–3.48 (d and m respectively, epoxy protons) and 4.08–4.38 (s, CH_2_ diglycidyl ether protons). ^13^C-NMR (DMSO-d_6_ ppm): δ_C_ 163.8 (CH=N), 161.72, 156.37, 154.44, 139.87, 135.64, 132.34, 130.48, 128.99, 126.92, 125.32, 117.23, 105.02 (aromatic carbons), 54.5 (-OCH_3_) and 50.52–45.53 (oxirane ring). Elemental analysis: found: C, 69.50; H, 5.67; N, 4.66, C_36_H_36_N_2_O_8_. Calcd: C, 69.20; H, 5.81; N, 4.49.

*4,4’-di(2,3-Epoxypropoxy-N-benzylidine-o-dianisidine* (**IIa**). The yield of yellow powder obtained was 75%. FT-IR (KBr disc): 1,621 cm^−1^ (CH=N stretch), 1,509 and 1,582 cm^−^^1^ (aromatic C=C stretch), 1,244 and 910 cm^−1^ (oxirane ring). ^1^H-NMR (CDCl_3_, ppm): δ_H_ 8.33 (s, CH=N), 7.75, 7.69, 7.63, 7.23 and 7.19 (s, d, d, d and d aromatic protons, respectively), 2.9–3.05 and 3.49–3.57 (d and m respectively, epoxy protons), 3.86 (s, -OCH_3_), and 4.02–4.40 (s, CH_2_ diglycidyl ether protons). ^13^C-NMR (DMSO-d_6_ ppm): δ_C_ 161.25 (CH=N), 159.83, 155.72, 138.20, 134.52, 130.02, 129.42, 125.01, 124.85, 118.00, 114.00, (aromatic carbons) and 55.44 (-OCH_3_) and 51.03–44.63 (oxirane ring). Elemental analysis: found: C, 72.08; H, 5.59; N, 5.11, C_34_H_32_N_2_O_6_. Calcd: C, 72.31; H, 5.72; N, 4.96.

*3,3’-Dimethoxy-4,4’-di(2,3-epoxypropoxy-N-benzylidine)-o-tolidine* (**IIIa**). A yellowish powder was obtained in 75% yield. FT-IR (KBr disc): 1,622 cm^−1^ (CH=N stretch), 1,511 and 1,578 cm^−1^ (aromatic C=C stretch), 1,269 and 914 cm^−1^ (oxirane ring). ^1^H-NMR (CDCl_3_, ppm): δ_H_ 8.34 (s, CH=N), 7.75, 7.63, 7.54, 7.31, 7.1 and 7.05 (s, d, d, d, s and d aromatic protons, respectively), 2.50 (s, Ph-CH_3_), 2.78–3.05 and 3.40–3.50 (d and m respectively, epoxy protons), 4.15–4.45 (s, CH_2_ diglycidyl ether protons) and 4.05 (s, -OCH_3_). ^13^C-NMR (DMSO-d_6_ ppm): δ_C_ 159.78 (CH=N), 159.28, 154.33, 137.73, 133.74, 132.94, 131.59, 128.50, 127.98, 127.63, 124.31, 118.00, 110.11 (aromatic carbons), 55.80 (-OCH_3_) and 50.49–44.69 (oxirane ring) and 18.5 (ph-CH_3_). Elemental analysis: found: C, 72.54; H, 6.11; N, 4.36, C_36_H_36_N_2_O_6_. Calcd: C, 72.94; H, 6.13; N, 4.73.

*4,4’-di(2,3-Epoxypropoxy-N-benzylidine)-o-tolidine* (**IVa**). Yellowish powder was produced in a yield of 74%. FT-IR (KBr disc): 1,624 cm^−1^ (CH=N stretch), 1,508 and 1,572 cm^−1^ (aromatic C=C stretch), 1,247 and 915 cm^−1^ (oxirane group). ^1^H-NMR (CDCl_3_ ppm): δ_H_ 8.39 (s, CH=N), 7.83, 7.5, 7.45, 7.2 and 6.73 (s, d, d, d and d aromatic protons, respectively), 2.50 (s, Ph-CH_3_), 2.78–2.95 and 3.48–3.59 (d and m respectively, epoxy protons) and 3.76–3.87 (s, CH_2_ diglycidyl ether protons). ^13^C-NMR (DMSO-d_6_ ppm): δ_C_ 159.80 (CH=N), 158.22, 139.95, 137.82, 132.49, 130.27 128.34, 127.75, 126.48, 122.28, 115.55 (aromatic carbons), 50.63–41.52 (oxirane ring) and 19.7 (ph-CH_3_). Elemental analysis: found: C, 76.15; H, 5.91; N, 4.97, C_34_H_32_N_2_O_4_.Calcd: C, 76.69; H, 6.06; N, 5.26.

*3,3’-Dimethoxy-4,4’-di(2,3-epoxypropoxy-N-benzylidine)-1,2-diaminoethane* (**Va**). A brownish powder was obtained in 76% yield. FT-IR (KBr disc): 1,608 cm^−1^ (CH=N stretch), 1,516 and 1,590 cm^−1^ (aromatic C=C stretch), 1,252 and 913 cm^−1^ (oxirane ring). ^1^H-NMR (CDCl_3_ ppm): δ_H_ 8.56 (s, H-C=N), 7.79 and 6.85 (d and d aromatic protons), 2.91–3.04 and 3.44–3.56 (d and s, epoxy protons), 3.92 (s, -OCH_3_) and 4.02–4.16 (s, CH_2_ diglycidyl ether protons). ^13^C-NMR (DMSO-d_6_ ppm): δ_C_ 164.4 (CH=N), 157.33, 154.23, 130.16, 124.41, 118.31, 109.03, (aromatic carbons), 55.8 (O-CH_3_), 50.80–40.89 (oxirane ring) and 28.39 (N-CH_2_). Elemental analysis: found: C, 65.13; H, 6.20; N, 6.14, C_24_H_28_N_2_O_6_. Calcd: C, 65.43; H, 6.41; N, 6.36.

*4,4’-di(2,3-Epoxypropoxy-N-benzylidine)-1,2-diaminoethane* (**VIa**). A brownish powder was obtained in 74% yield. FT-IR (KBr disc): 1,610 cm^−1^ (CH=N stretch), 1,516 and 1,589 cm^−1^ (aromatic C=C stretch) 1,250 and 912 cm^−1^ (terminal oxirane). ^1^H-NMR (CDCl_3_ ppm): δ_H_ 8.32 (s, CH=N), 7.4, 7.26 and 7.03 (s, d and d aromatic protons, respectively), 4.5–3.8 (s, CH_2_ diglycidyl ether protons) and 3.55–3.42 and 3.04–2.79 (m and d respectively, epoxy protons). ^13^C-NMR (DMSO-d_6_ ppm): δ_C_ 162.0 (CH=N), 159.60, 128.83, 125.05, 117.00 (aromatic carbons), 51.25–41.23 (oxirane ring) and 27.52 (N-CH_2_). Elemental analysis: found: C, 69.83; H, 6.58; N, 7.42, C_22_H_24_N_2_O_4_. Calcd: C, 69.44; H, 6.36; N, 7.37.

## 4. Conclusions

The targets of the synthesis of the compounds and determination of their properties were successfully achieved. The phenolic Schiff bases and diglycidyl ethers were characterized by spectroscopy and elemental analysis (CHN). The diglycidyl ethers **Ia**–**IVa** were found to display enantiotropic nematic phases, based on the POM and DSC observations, whereas, **Va** and **VIa** melted without exhibiting any transition mesophases.
